# Coffee Extract as a Natural Antioxidant in Fresh Pork Sausage—A Model Approach

**DOI:** 10.3390/foods13091409

**Published:** 2024-05-03

**Authors:** Vanessa Tanara Fetsch, Daneysa Lahis Kalschne, Cristiane Canan, Éder Lisandro de Moraes Flores, Marcelo Caldeira Viegas, Gabrielle Caroline Peiter, Ricardo Fiori Zara, Joana Soares Amaral, Marinês Paula Corso

**Affiliations:** 1 Post-Graduate Program in Food Technology (PPGTA), Academic Department of Food, Federal University of Technology—Paraná, Campus Medianeira (UTFPR-MD), Avenida Brasil 4232, Medianeira 85722-332, Brazil; vanessatanara@gmail.com (V.T.F.); daneysakalschne@utfpr.edu.br (D.L.K.); corso@utfpr.edu.br (M.P.C.); 2 Academic Department of Chemistry, Federal University of Technology—Paraná, Campus Medianeira (UTFPR-MD), Avenida Brasil 4232, Medianeira 85722-332, Brazil; eder@utfpr.edu.br; 3 IGC—Companhia Iguaçu de Café Solúvel S.A., Research and Development, BR-369, Km 88, Cornélio Procópio 86300-000, Brazil; mviegas@iguacu.com.br; 4 Academic Department of Chemistry, Federal University of Technology—Paraná, Campus Toledo (UTFPR-TD) Rua Cristo Rei, 19, Toledo 85902-490, Brazil; gabriellepeiter@utfpr.edu.br (G.C.P.); ricardozara@utfpr.edu.br (R.F.Z.); 5 Centro de Investigação de Montanha (CIMO), Instituto Politécnico de Bragança, Campus de Sta. Apolónia, 5300-253 Bragança, Portugal; jamaral@ipb.pt

**Keywords:** *Coffea arabica*, *Coffea canephora*, caffeine, 5-caffeoylquinic acid, antioxidant activity, meat products

## Abstract

Consumers are increasingly looking for healthy foods without the addition of synthetic additives. The aim of this study was to evaluate the efficiency of coffee extracts as a natural antioxidant in fresh pork sausage. Firstly, the conditions for obtaining coffee green extracts were optimized (Central Composite Rotatable Design 2^3^, variables: extraction time, ethanol–water ratio, and sample–solvent ratio) in an ultrasound bath (70 °C). The response variables were the bioactive compounds levels and antioxidant activity. Valid models were obtained (*p* ≤ 0.05, R^2^ > 0.751), with higher bioactive content and antioxidant activity in the central point region. Extracts of Robusta and Arabica coffee green (RG and AG) and medium roast (RR and AR) obtained, and central point (10 min, an ethanol concentration of 30%, and a sample–solvent ratio of 10 g/100 mL) and optimized (14.2 min, 34.2%, and 5.8 g/100 mL) parameters were characterized. The RG presented a significantly (*p* ≤ 0.05) higher content of caffeine (3114.8 ± 50.0 and 3148.1 ± 13.5 mg/100 g) and 5-CQA (6417.1 ± 22.0 and 6706.4 ± 23.5 mg/100 g) in both extraction conditions, respectively. The RG and RR coffee presented the highest antioxidant activity. Two concentrations of RG and RR coffee extracts were tested in fresh pork sausage. The Robusta coffee extract presented the highest antioxidant activity in both roasted and green states. However, when applied to a meat product, the extract prepared with RG coffee showed better results, with efficiency in replacing synthetic antioxidants (content of malonaldehyde/kg of sample below 0.696 ± 0.059 in 20 days of storage), without altering the sensory attributes of the product (average scores above 7.16 ± 1.43 for all attributes evaluated). Therefore, the RG coffee extract was a suitable alternative as a natural antioxidant applied to fresh pork sausage.

## 1. Introduction

Antioxidants are natural or synthetic additives used to prevent lipid oxidation and increase food shelf life [1]. In the meat industry, for a long time, synthetic antioxidants (butylated hydroxyanisole (BHA), butylated hydroxytoluene (BHT), and terc-butylhydroquinone (TBHQ), sodium erythorbate, and propyl gallate (PG) were the most used. However, due to consumers’ growing desire for “clean label’s” foods, the food industry is focused on replacing the use of synthetic by natural antioxidants. To meet this demand, several studies have been carried out looking for different sources of natural antioxidants that are more efficient and economically viable [2].

Coffee is one of the most consumed drinks in the world, surpassed only by water [3], and its market prospects remain promising. World coffee production saw a marginal increase of 0.1% from 2022 to 2023, reaching 168.2 million bags. Although the world’s coffee consumption slightly declined by 2.0% to 173.1 million bags in the same period, it is expected to grow by 2.2% (to 177.0 million bags) in 2024 [4]. Its consumption is motivated mainly by a pleasant flavor and aroma, positive sensations, and physiological effects [5]. The main cultivated species are arabica (*Coffea arabica* L.) and canephora (*Coffea canephora*). Arabica coffee has a majority share in the global production of coffee beans [6], mainly because it is considered a more noble coffee, with a more intense and characteristic flavor due to a greater variety of polyphenols, while Robusta coffee has a unique flavor, is less acidic, and has a higher caffeine content, which makes it more bitter [7]. Previous studies have reported that coffee is rich in antioxidant compounds such as phenolic compounds, especially chlorogenic acids and their degradation products (caffeic, ferulic, and coumaric acids), as well as antioxidants such as caffeine, melanoidins, and trigonelline [5,8,9]. Coffee antioxidant capacity varies according to the species and is affected by post-harvest processing conditions (drying, storage, roasting, and grinding processes) and by the extraction method [10]. The roasting process is responsible for giving the brew its characteristic properties, such as color, flavor, and aroma, and considerably alters the chemical composition of the coffee. While natural phenolic compounds may be lost, other antioxidant compounds are formed as the products of the Maillard reaction (PRMs), as melanoidins, furfural, and hydroxymethylfurfural, in addition to apparent color change [7,11].

A variety of extraction methods can be used for brew elaboration. When considering the extraction of coffee antioxidants for use as additives in the food industry, the most common are Soxhlet, conventional solid–liquid, and liquid–liquid extractions. However, these methods are associated with a higher risk of degradation of thermally unstable compounds, greater solvent consumption, and a longer extraction time [1,12]. Therefore, to obtain extracts with higher yield, less degradation of bioactive compounds, and lower cost, other extraction methods are being studied, among which ultrasound-assisted extraction (EAU) may be highlighted. This technique presents promising results, showing improved yield in the extraction of bioactive compounds, with reduced extraction time and smaller solvent volume compared to other methods [12,13,14]. The principle of the ultrasound technique is based on the cavitation effect. In this phenomenon, waves formed by ultrasound, transmitted at a frequency higher than human hearing capacity, pass through the medium, creating compression and expansion, which generates a process in which bubbles are produced, grow, and collapse [13]. The collapse of the bubbles results in the rupture of the cell walls, facilitating mass transfer [15]. Among the solvents suitable for UAE, ethanol stands out due to its low cost, low environmental impact, and status as a renewable solvent categorized as GRAS (generally recognized as safe). Ethanol presents a great affinity for phenolic compounds [13], and has already been successfully used in extracting bioactive compounds from coffee, both in pure form [16,17] and in mixtures of ethanol and water [18].

The extraction method, coffee species, roasting degree, and extraction conditions directly influence the bioactive extract obtained. Moreover, there is little information available regarding ultrasound-assisted extraction effect on coffee bioactive content. Therefore, the present study aimed to obtain a coffee extract with a high bioactive content and antioxidant activity by optimizing the extraction conditions, and additionally evaluating the effectiveness of this natural antioxidant in a fresh pork sausage, comparing its sensory acceptance compared to that of a synthetic antioxidant.

## 2. Materials and Methods

**Figure 1:**
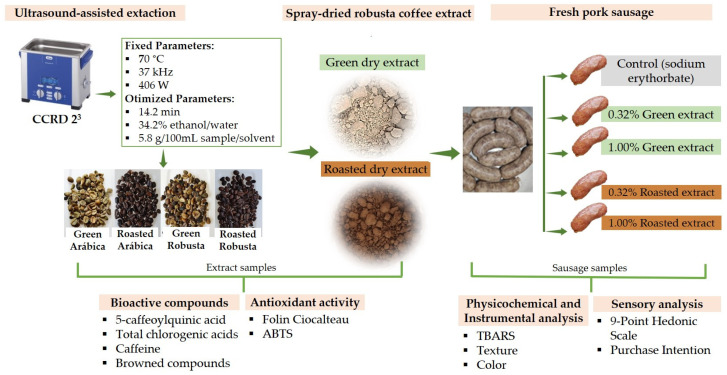
Work flow chart: obtaining dry green coffee extract and application in fresh pork sausage.

An experiment flow schematic with the main steps for obtaining dry coffee extract and application in fresh pork sausage is shown in Figure 1.

### 2.1. Materials and Characterization

To optimize the extraction conditions (by the central composite rotational design (CCRD)), a commercial Arabica coffee medium roast and grounded (AC) was used. For evaluation of different coffee samples under the optimized extraction conditions, Robusta coffee green (RG) and medium roast (RR) and Arabica coffee green (AG) and medium roast (AR) were used, supplied by IGC—Cia Iguaçu de Café Solúvel (Cornélio Procópio, Paraná, Brazil). The samples were ground (IKA, A11, Staulfen, Germany) using a similar mesh to that for commercial coffee. All coffee samples were characterized by granulometry, using sieve sizes 16, 20, 30, 35, and 60 mesh and electromagnetic sieve shaker at level 6 for 15 min (Bertel, Caieiras, Brazil); by color parameters L* (lightness; 100 = white, 0 = black), a* (redness; +, red; −, green), and b* (yellowness; +, yellow; −, blue), determined with the CIELAB system using a colorimeter (CR 400, Konica Minolta, Osaka, Japan) calibrated with a D65 standard illuminant and a 10° angle; and by moisture [19]. All reagents were of analytical grade.

### 2.2. Ultrasound-Assisted Extraction (UAE) and Optimization Procedure

Coffee extracts were prepared by coffee dissolution in an ethanol–water solution and submitted to an ultrasound bath (Elmasonic P, Elma, Singen, Germany) at 70 °C, 37 kHz frequency, and 406 W potency (method and conditions based on previous tests. The coffee extracts were vacuum filtered (qualitative filter paper 80 g/m^2^) and stored frozen in an amber glass bottle.

To optimize the extraction procedure, a central composite rotational design 2^3^ (CCRD) including 6 axial points and 3 repetitions in the central point, totalizing 17 runs, was performed [20]. The independent variables were the ultrasound exposure time (X_1_; 1.6 to 18.4 min), ethanol concentration (X_2_; 21.6 to 38.4%), and coffee–solvent ratio (X_3_; 1.6 to 18.4, 1.6 to 18.4 g/100 mL), while the dependent variables were the content of browned compounds (BC; Y_1_), caffeine (CAF; Y_2_), 5-caffeoylquinic acid (5-CQA; Y_3_), total chlorogenic acids (CGA; Y_4_), and antioxidant activity determined by Folin–Ciocalteau (FC; Y_5_) and ABTS+• methodologies (ABTS+•, Y_6_).

### 2.3. Analysis of Bioactive Compounds and Antioxidant Activity

Browned compounds (melanoidins) were estimated according to Ludwig et al. (2012) and Marcucci et al. (2013) [21,22]. Coffee extracts were diluted in ultrapure water up to 0.57 mg/mL and the absorbance was determined at 420 nm (PerkinElmer, Lambda XLS, Beaconsfield, UK). The absorbance value was considered indicative of the formation of browned compounds in coffee roasting, and as an estimate of melanoidins. The results are expressed in AU.

The caffeine, 5-caffeoylquinic acid, and total chlorogenic acids were determined according to Dias and Benassi (2015) and Kalschne et al. (2019) [23,24] using an ultra-high performance liquid chromatograph (UPLC) (Ultimate 3000, Thermo Scientific, Germering, Germany), equipped with an automatic sample injector, quaternary pump, oven, and diode array (DAD) detector, and controlled by Chromeleon 7.0 software. Coffee extracts were diluted in acetic acid:water (5:95 *v*/*v*) solution up to 0.003 g/mL, filtered (0.45 µm, Millipore, Burlington, MA, USA), and injected (20 μL) into the chromatograph. The column used was a Hypersil Gold^TM^ (Thermo Fischer Scientific, Waltham, MA, USA) (150 mm × 4.6 mm, 3 μm particle size). The mobile phase was composed of acetic acid/ultrapure water (5:95 *v*/*v*) (A) and acetonitrile (B), and the following gradient elution was used: 1 min, 5% B; 5 min, 13% B; flow rate of 0.6 mL/min. Detection was set at 272 nm for caffeine and 320 nm for chlorogenic acids. For UPLC analysis, the identification of the compounds was based on the retention times and UV spectra. Quantification was performed by external standardization using 6-point analytical curves with triplicate measurements (R^2^ ≥ 0.999 and *p* < 0.001). The concentration ranges were 5 to 65 μg/mL for caffeine and 0.5 to 30 μg/mL for 5-CQA. The total chlorogenic acid (CGA) content was estimated by the sum of areas of detected compounds at 320 nm based on Corso et al. (2016) [25] using 5-CQA as a standard for quantification. All results are expressed on dry basis as mg of a compound/100 g of coffee.

The antioxidant activity was determined by the Folin–Ciocalteau indirect method carried according to Vignoli et al. (2011) [5]. Solutions with five known concentrations of gallic acid in the range of 0.07 to 0.48 g/L were used for calibration (R^2^ = 0.99). The results are expressed in g of gallic acid equivalent (GAE)/100 g of sample on a dry basis. The hydrogen ions donation activity to the ABTS radical (ABTS+•) was performed [26]. Ethanol solutions with six concentrations of Trolox in the range of 0.1 to 0.8 mMol were used for calibration (R^2^ = 0.99). Results are expressed as antioxidant capacity equivalent to Trolox (TEAC) in g of Trolox/100 g of sample in dry basis.

### 2.4. Response Surface Analysis, Desirability and Experimental Validation

The dependent variables are expressed by mean ± standard deviation (*n* = 3). The CCRD was analyzed using the Experimental Design procedure of Statistica 8.0 software. The adequacy of the models generated by CCRD was assessed, and dependent variables with significant effects (*p* ≤ 0.05), R^2^ ≥ 0.751, and adjusted R^2^ ≥ 0.557 were considered. To optimize extraction conditions, the global desirability function was determined using the Response Desirability Profiling procedure of Statistica 8.0. The goal was to maximize the content of the bioactive compounds and antioxidant activity. For the experimental validation of CCRD mathematical models, commercial coffee extraction was performed at the optimized extraction condition. The experimental results were compared with the predicted ones.

### 2.5. Different Coffee Samples Extraction under Optimized Conditions and Spray-Drying Process

Different coffee samples comprising green or roasted Arabica or Robusta were extracted under the optimized conditions that were determined with a commercial roasted and ground Arabica coffee (as described in Section 2.2). The RG, RR, AG, and AR coffee extracts were characterized for their bioactive compounds content and antioxidant activity, as performed for the extracts obtained during CCRD runs (Section 2.3). The coffee samples that provided a higher bioactive content and antioxidant activity were selected and spray-dried (MSD 1.0, Labmaq, Ribeirão Preto, Brazil). The coffee extract feeding and drying process was carried out using a peristaltic pump at drying air pressure of 2 kgf/cm, drying air flow of 25 L/min, drying air flow of 55 Nm^3^/h, 1 mm diameter injection nozzle, 90 °C drying chamber, and 300 mL/h flow rate to obtain coffee extract powdered. Drying parameters were based on previous tests. The dried coffee extracts were characterized for their bioactive compounds and antioxidant activity as previously described. A sample of commercial antioxidant based on sodium erythorbate was also evaluated for comparison and determination of the amount to be tested in the meat product.

### 2.6. Application of Dry Coffee Extracts as Natural Antioxidants in a Fresh Pork Sausage

The preparation of the pork sausage product followed the prescribed regulations concerning ingredients [27], and the process described by Viera et al. (2016) [28], with modifications. Spray-dried extracts of green (GDE) and roasted (RDE) Robusta coffee were applied to a conventional fresh pork sausage (Tuscan-style) formulation (pork meat 91.0%, ice water/ice 6.0%, salt 2.0%, curing salts 0.2%, condiment 0.5%, garlic powder 0.1%, white pepper 0.02%, monosodium glutamate 0.1%, oregano 0.02%, parsley 0.02%), in two concentrations, 0.32% and 1%, totaling four formulations (GDE1%, GDE032%, RDE1%, and RDE032%). The formulations were compared with a control (C), made with the same ingredients but containing the commercial synthetic antioxidant sodium erythorbate (0.08%).

A local pig slaughterhouse with a federal inspection service supplied the meat raw material used to prepare the samples. The cold meat was weighed and ground in a cutter (MADO, Garant MTK 661, Dornhan, Germany), and mixed manually with the other previously weighed ingredients (except the antioxidant). The obtained meat mass (15 kg) was divided into 5 parts, and the appropriate amount of coffee extract or commercial antioxidant was added to each of them (GDE1%, GDE032%, RDE1%, RDE032%, and C). The dough was homogenized manually, stuffed into a 30 mm natural pork casing using a filling machine (IV20, series V195001, RB engineering, Seriate, Italy), and manually tied into segments of approximately 10 cm. Afterward, the samples were vacuum packed (Microvac CV8, Selovac, São Paulo, Brazil) and stored under refrigeration at 2 ± 2 °C.

### 2.7. Physicochemical, Sensory Acceptance, and Instrumental Evaluation

The moisture, ash, protein, carbohydrate, and lipid content of the fresh pork sausage base formulation was determined in duplicate following the Official Methods of Analysis of the Association of Analytical Chemists (AOAC) [19].

Lipid oxidation was determined in triplicate after the preparation of the sausages (1 day) and after 10 and 20 days of storage under refrigeration according to Tarladgis, Pearson and Dugan (1964) [29] and subsequent modifications [30]. The samples (100 g) were crushed in a grinder (IKA, A11, Staulfen, Germany) and 10 g of the homogenized sample was weighed on an analytical balance (Marte, AW220, São Paulo, Brazil). The absorbances were read using a UV–Vis spectrophotometer (PerkinElmer, Lambda XLS, Beaconsfield, UK) at 530 nm. A standard curve (R^2^ = 0.99) was prepared using a solution of 1,1,3,3-tetraethoxypropane (TEP) in deionized water at concentrations of 1 × 10^−7^ to 6 × 10^−6^ Mol/L of TEP. The analysis recovery percentage was 92%. The results are expressed in mg of malonaldehyde/kg of sample.

The sensory evaluation of the samples was carried out after approval by the Human Research Ethics Committee of the Federal Technological University of Paraná (CAAE 29497820.9.0000.5547, certificate no. 4.008.703) and after ensuring the microbiological quality of the samples though adequate analysis testing. Seven days after being prepared, the five samples were baked on a barbecue stove, over medium heat for 40 min until an internal temperature of 74 °C was achieved. Then, they were served monadically in 30 g portions coded with 3 random digits for each consumer, following a balanced complete block design. The samples were evaluated by 62 consumers of meat products (male = 42%, female = 58%), aged between 18 and 35 years (73%) and 36 and 65 years (27%) using a 9-point hedonic scale (1 = extremely disliked and 9 = extremely liked) to evaluate the attributes color, smell, flavor, texture, and overall acceptance, and a 5-point scale (5-point structured scale (1 = would certainly not buy and 5 = would certainly buy) to evaluate purchase intention. The model of informed consent form collected from consumers can be consulted.

The parameters of the samples—hardness, elasticity, chewiness, and cohesiveness—were evaluated in triplicate, using a texture analyzer (Stable Micro Systems, TA.HD Plus, Vienna Court, UK). The samples (baked as described) were cooled to room temperature and subsequently cut into cylinders 2 cm thick in diameter, and the casings were removed. They were then compressed in two consecutive compression cycles with a 45 mm diameter probe at a pre-test speed of 1 mm/s, test speed and post-test speed of 5 mm/s, distance of 10 mm, time of 5 s, and trigger force of 5 g, compressing 50% of the sample height. Instrumental color evaluation was carried out in quintuplicate on both raw and baked samples, using a Minolta CR 400 colorimeter with D65 illuminant and 10° viewing angle. The values of L* (brightness), a* (red–green component), and b* (yellow–blue component) are expressed in the CIELAB color system.

### 2.8. Data Analysis

Physicochemical and instrumental data were evaluated by analysis of variance (ANOVA) (one-way) and sensory data by ANOVA (two-way), followed by Tukey’s mean comparison test (*p* ≤ 0.05). Statistical analysis was performed using the Statistica 8.0 program (Statsoft Inc., Tulsa, OK, USA).

## 3. Results and Discussion

### 3.1. Obtaining Coffee Extract for Use as a Natural Antioxidant: CCRD Approach

First, the extraction was performed using Arabica commercial coffee (AC) medium roast (L = 20.65 ± 0.08, a* = 8.05 ± 0.11 and b* = 16.31 ± 0.22), with granulometry of 0.84 to 1.00 mm and moisture 4.68 ± 0.14 g 100 g^−1^. The obtained bioactive compounds content and antioxidant activity of CCRD coffee extracts (Table 1) are in the expected range.

Browned compounds (melanoidins) varied from 0.236 to 0.778 UA; caffeine ranged from 1241.14 to 1502.14 mg/100 g, 5-caffeoilquinic acid ranged from 123.14 to 269.66 mg/100 g, and total chlorogenic acids ranged from 503.90 to 1309.40 mg/100 g. Regarding antioxidant activity, the ABTS+• ranged from 6.61 to 21.37 g Trolox/100 g and via Folin–Ciocalteau methods, variations were found between 4.14 and 7.22 g EAG/100 g. Vignoli et al. (2011) [5] studied soluble coffees of the Arabica type and reported that melanoidin levels varied depending on the coffee species and extraction process but were most affected by the degree of roasting (light 20.13 ± 0. 59, medium 22.08 ± 0.04, and dark 25.41 ± 0.01 g melanoidins/100 g). According to Almeida and Benassi (2008) [31] the melanoidin values of commercial roasted coffees displayed considerable variability, ranging from 0.291 to 0.690 of 0.291 to 0.690 in absorbance measured at 420 nm, indicating the likelihood of significant differences occurring in the roasting processes used. Additionally, absorbance values of 0.253 and 0.476 were observed for regular coffees and an average value of 0.330 for decaffeinated coffees. Overall, the increase in the melanoidin content occurs with the evolution of the degree of roasting, due to the development of the Maillard reaction [22].

5-caffeoilquinic acid (1.39 to 1.96 g/kg dry wt) was the most abundant phenolic compound in all green coffee samples analyzed, followed by chlorogenic acid (0.19 to 0.49 g/kg dry wt). Caffeine (0.79 to 1.84 g/kg dry wt) was the second most abundant compound in all analyzed samples [32]. The highest values for total phenolic compounds (TPC) were found in coffee extracts from *C. canephora* (0.388 ± 22.43 g GAE/100 g), while *C. arabica* had a TPC of around 30% smaller than *C. canephora* (0.280 ± 28.72 g GAE/100 g). Regarding scavenging activity (ABTS+•), it was generally higher in *C. canephora* and was associated with TPC content [33]. Table 2 details the effects of ultrasound exposure time, ethanol concentration, and coffee–solvent ratio on browned compounds, caffeine, 5-caffeoylquinic acid, total chlorogenic acids, and antioxidant activity.

The browned compounds had a positive effect on the mean, x_2_, and interaction x_2_x_3_, while they had a negative effect on x_3_ and x_2_^2^. This suggests that an increase in ethanol concentration and a reduction of sample–solvent ratio improves the browned compounds extraction. The caffeine had a positive effect on the mean and negative effect on x_3_ and x_3_^2^, suggesting that a decrease in the sample–solvent ratio tends to improve caffeine extraction. The 5-CQA and TCA showed a negative effect on the mean of x_1_^2^, x_2_^2^, and x_3_^2^; a decrease in ultrasound exposure time, ethanol concentration, and sample–solvent ratio tends to improve this bioactive content. The FC had a positive effect on mean, x_2_, and x_3_^2^, while it had a negative effect of x^3^. An increase in ethanol concentration and a decrease in sample–solvent relation improves antioxidant activity. The ABTS+• had a positive effect on mean x_3_^2^ and a negative effect on x_3_.

**Figure 2:**
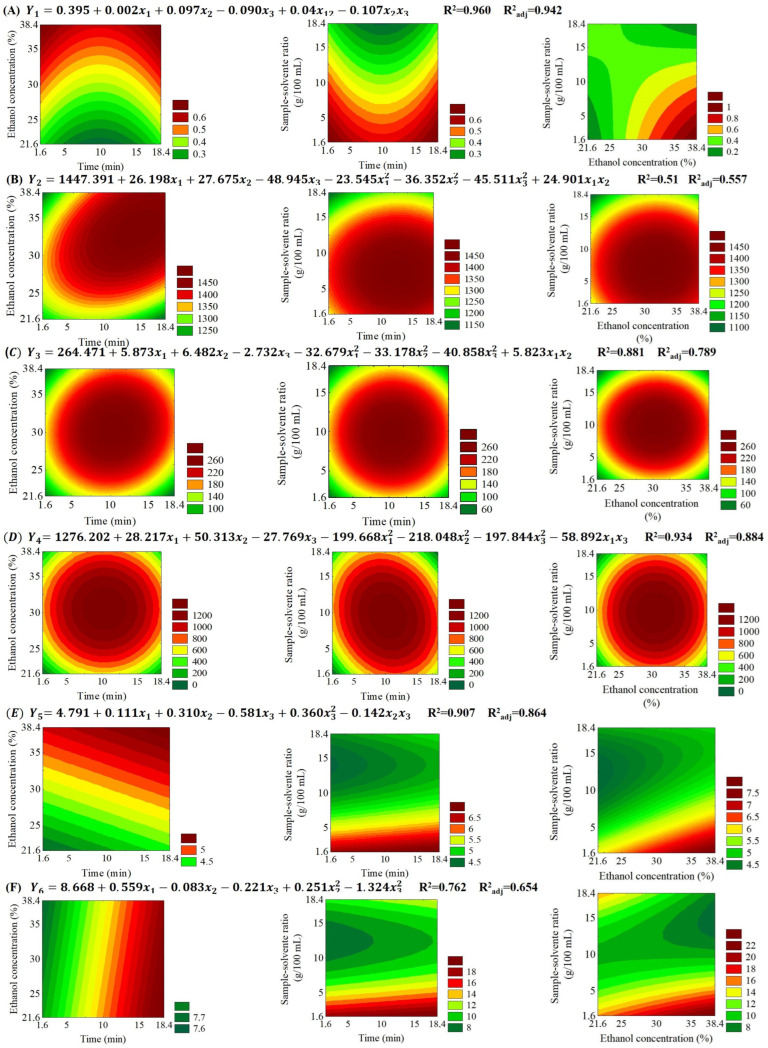
Response surface of (**A**) browned compounds, (**B**) caffeine, (**C**) 5-caffeoylquinic acid, (**D**) total chlorogenic acids, (**E**) antioxidant activity via Folin–Ciocalteau and (**F**) antioxidant activity via ABTS+• radical. Y_1_ denotes browned compounds; Y_2_, caffeine; Y_3_, 5-caffeoilquinic acid, Y_4_, total chlorogenic acids, Y_5_, total phenolic compounds, Y_6_, ABTS•+ scavenging activity, x_1_, ultrasound exposure time, x_2_, ethanol concentration, and x_3_, sample–solvent ratio.

Valid quadratic models (with R^2^ ≥ 0.75 and adjusted R^2^ ≥ 0.56) were obtained for all bioactive compounds and antioxidant activity. Non-significant effects were incorporated into the residue of the models, except in cases in which the effect influenced negatively the adjusted R^2^. Corresponding models and surface responses are shown in Figure 2; the ANOVA of models is detailed in Table 3.

The response surfaces A, E, and F (Figure 2) show that an optimal extraction point was not obtained in the studied range. Still, it is possible to verify that the higher the proportion of ethanol in water and the lower the sample–solvent ratio, the greater the number of browned compounds, reducing activity content and hydrogen ion donating activity to the ABTS+• radical obtained, which shows that in the range studied, there was not enough solvent for the maximum extraction of compounds that could be extracted. Surfaces B, C, and D show that the extraction of caffeine, 5-CQA, and total chlorogenics was optimized at the central point.

A desirability analysis was carried out to define the best extraction parameters, seeking to obtain the best antioxidant activity and assess the evaluated antioxidant compounds, which allowed for obtaining the optimal values of the investigated parameters.

**Figure 3:**
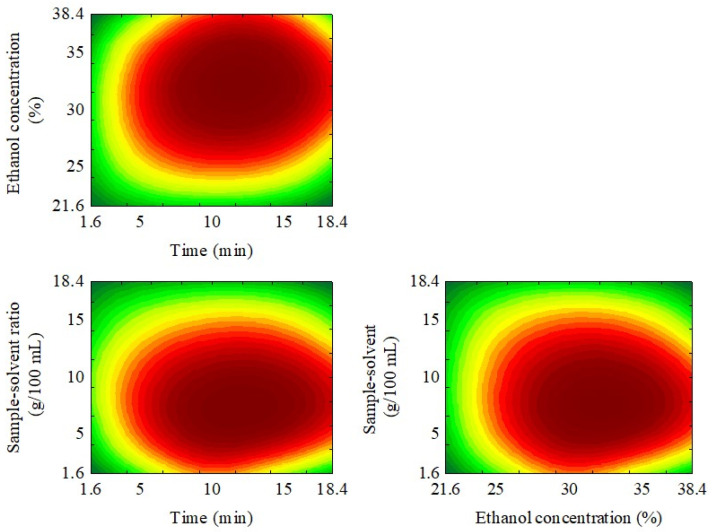
Desirability graph showing the optimum region to obtain higher content of bioactive compounds and high antioxidant activity.

Figure 3 shows the desirability graph in order to obtain the greater content of bioactive and antioxidant activity.

The desirability graphs suggest that an improved extraction procedure can be achieved by employing an ultrasound exposure time of 14.2 min, an ethanol concentration of 34.2%, and a sample–solvent ratio of 5.8 g/100 mL. Under optimized conditions, the predicted values were 0.666 UA for browned compounds, 1492 mg/100 g for caffeine, 205 mg/100 g for 5-CQA, 1004 mg/100 g for TCA, 6.04 g EAG/100 g for FC, and 13.47 g Eq Trolox/100 g for ABTS+• (all dry matter (d. m.)).

After determining the optimized conditions, the models were validated and the caffeine content (mg/100 g d. m.), FC (g EAG/100 g d. m.), and ABTS+• (g Eq Trolox/100 g d. m.) at the optimized point (1654 ± 109, 5.64 ± 0.08 and 14.90 ± 0.91, respectively) were greater than those obtained at the center point (1364 ± 51, 4.74 ± 0.07 and 10.89 ± 0.54) (*p* < 0.05). Caffeine content and antioxidant activity via FC and ABTS+•, at the optimized point, represent 110%, 93%, and 111% of predicted value, while at the center point they represent 91%, 78%, and 81%. The content of 5-CQA was similar at the optimized point (327 ± 16) and central point (315 ± 21) and represented 159% and 154% of the predicted value.

Because an optimal extraction region was found in the central point region, new extractions were performed using different coffee samples (Robusta and Arabica roasted coffee, and Robusta and Arabica green coffee) under these parameters for comparison with the optimized point (Table 4).

The coffee color parameters suggest that roasted samples had a medium roasting degree [34], although Robusta coffee (L* = 30.26 ± 0.43, a* = 9.38 ± 0.08, b* = 23.22 ± 0.22, moisture 4.36 ± 0.04 g 100 g^−1^) was lighter than Arabica coffee (L* = 22.27 ± 0.30, a* = 8.86 ± 0.05, b* = 16.85 ± 0.12, moisture 5.40 ± 0.07 g 100 g^−1^). The green coffees (Arabica: L* = 56.12 ± 0.14, a* = −0.25 ± 0.3, b* = 23.01 ± 0.04, moisture 11.11 ± 0.07 g 100/g, and Robusta: L* = 55.65 ± 0.15, a* = −0.71 ± 0.14, b* = 24.16 ± 0.27, moisture 9.30 ± 0.11 g/100 g) had a similar L* parameter (*p* > 0.05). The moisture of the green and roasted samples showed a significant difference (*p* ≤ 0.05), with green coffees, as expected, having higher moisture.

The green Robusta coffee sample had a significantly (*p* ≤ 0.05) higher caffeine and 5-CQA content, followed by green Arabica coffee. This may be explained by the lack of a roasting process, which changes coffee bean composition and destroys part of its bioactive compounds [7,11]. It was observed that despite caffeine being more thermally stable than 5-CQA, a large part of caffeine was lost in the roasting process. There was no significant difference (*p* > 0.05) between extraction at the optimum point and at the central point in obtaining 5-CQA, and in the case of caffeine, there was a difference only for roasted coffees, where the extraction was more efficient at the optimal point. The cases without significant differences are in accordance with response surfaces, which suggest the existence of an optimal extraction region and not just one point.

It is possible to verify that the extracts with the highest antioxidant activity, under the parameters employed in this study, were the green and roasted Robusta coffees, both in terms of the extraction at the optimum point and in the central point.

Overall, it was demonstrated that selecting the appropriate coffee species to be used as raw material for extraction, together with the optimization of extraction parameters within an optimal region, enable the production of extracts with higher bioactive potential to be used as an additive as an alternative to synthetic antioxidants in raw meat products. Finally, both roasted and green Robusta coffee extracts obtained at the optimum point presented the most promising results of antioxidant activity (Table 4).

### 3.2. Application of Coffee Extract as a Natural Antioxidant in Fresh Pork Sausage: Oxidative Stability, Physicochemical, and Sensory Properties

The base formulation of the fresh pork sausage had a chemical composition of 56.12 ± 0.43% moisture, 13.02 ± 0.03% proteins, 26.54 ± 0.20% lipids, 1.68 ± 0.02% carbohydrates, and 3.42 ± 0.17% ash. The TBARS values of the fresh pork sausage samples prepared with green and roasted Robusta coffee extract, as a natural antioxidant, and the control formulation with a commercial antioxidant are shown in Table 5.

It is possible to observe that there was no significant difference (*p* > 0.05) in the malonaldehyde content of the samples and that there was no significant increase during the storage time evaluated (Table 5). The values varied between 0.612 ± 0.056 and 0.697 ± 0.065 mg of malonaldehyde/kg of sample. Previous studies suggest that, in sausages, the TBARS threshold for consumer detection of rancidity is 1.0 mg/kg [34]. Thus, the obtained results indicate that the antioxidant activity of coffee extracts obtained from both green and roasted Robusta coffee, at the tested concentrations, was efficient in controlling lipid oxidation in sausage samples until the 20th day, similarly to sodium erythorbate (0.08%).

Dilnawaz et al. (2017) [35] evaluated the use of 1% Arabica green coffee extract in restructured blocks of mutton and obtained similar TBARS values up to the 14th day of the study. Jully et al. (2016) [36] evaluated the antioxidant activity of different coffee roasts, in the grounds and freeze-dried forms, and found that the addition of coffee neither inhibited nor promoted the oxidation of proteins in cooked hamburgers, but inhibited lipid oxidation, resulting in values comparable to those of pork with the addition of rosemary oleoresin. Lin et al. (2015) [37] also found results showing that roasted ground coffee can extend the shelf life of unsalted and salted raw meats, working as effectively or better than rosemary oleoresin as they found lower malonaldehyde values compared to the control without added antioxidants.

However, aiming for an application of this natural ingredient to a meat product, it is essential that it not only offers technological advantages, but also does not lead to sensory depreciation of the product. The fresh pork sausage prepared with 0.32% spray-dried extract of green Robusta coffee (GDE032%) showed no significant difference (*p* > 0.05) from the control sample for the attributes smell, color, flavor, texture, and overall acceptance, with scores above 7.43 ± 1.16, (referring to the scale: 7 = I liked it moderately and 8 = I liked it a lot) and purchase intention above 4.21 ± 0.86 (referring to the scale: 4 = I would probably buy it and 5 = I would certainly buy it), indicating the potential for use of the extract as a natural antioxidant ingredient (Table 5). For the texture attribute, only the sample prepared with 1% roasted coffee extract (RDE1%) was negatively affected. However, for the other attributes, the samples GDE1%, RDE032%, and RDE1% had a decrease (*p* < 0.05) in acceptance, therefore limiting the use of the GDE extract to 0.32%, considering the product and concentrations tested. This may differ for different meat products and formulations; for example, Dilnawaz et al. (2017) [35] obtained promising sensory results even when they added 1% green coffee extract to blocks of restructured mutton.

The instrumental texture analysis showed that the use of coffee extract at the tested concentrations did not interfere with the hardness, elasticity, or chewiness of the fresh pork sausage samples (*p* > 0.05) (Table 5). However, there was a reduction in the cohesiveness parameter with the addition of roasted coffee, especially in the sample added at 1%. This may also justify the lower sensory acceptance (*p* < 0.05) for this sample in the texture attribute. According to Cao et al. (2015) [38], meat processed with spices and herbs rich in polyphenols can have its texture altered. The authors found that the addition of 150 µmol/g of chlorogenic acids influenced oxidation-induced changes in the gelling properties of the myofibrillar protein. Although the green coffee extract has more 5-CQA than the roasted extract (Table 4), it can be observed using the Folin–Ciocalteau method that the total phenolic content in roasted Robusta is still high. Research shows that among the various changes that occur during roasting, one of the major changes regards the composition of phenolic compounds. The content of chlorogenic acids falls because their isomerization occurs, leading to an increase in 3- and 4-caffeoylquinic acids, and giving rise to various isomers of caffeoylquinic lactones, ferulylquinic lactones, and caffeoylquinic acids [7,39].

As expected, the colorimetric analysis showed that green coffee extracts caused less color change in the meat product, both in the raw product and after cooking. Similarly, in both the raw and cooked samples, prepared with 0.32% green coffee extract, there was no significant difference for all color parameters (L*, a*, and b*) (*p* > 0.05), corroborating the sensory results. On the contrary, in the samples made with the roasted coffee extract, the product was visually darkened due to the brown color of the extract, which was confirmed by the lower L* values (*p* < 0.05).

It is noteworthy that in the present work it was not possible to evaluate the interaction of antioxidant compounds from spices and added extracts. However, it would be interesting to evaluate this interaction carried out on model sausages, as well as to evaluate the effects of extracts on other meat products with different processes such as cooking and fermenting, among others.

## 4. Conclusions

Using a CCRD approach, an optimal extraction region within the studied range was identified, where a higher content of bioactive compounds and consequently higher antioxidant activity was achieved in the region of the central point with optimization of responses under conditions of 14.2 min extraction time, 34.2% ethanol–water ratio, and 5.8 g/100 mL sample–solvent ratio in ultrasound-assisted extraction. By applying the optimized extraction conditions to Robusta and Arabica coffees, the former evidenced the highest antioxidant activity in both the medium roasted and green states. However, when applied to a meat product (fresh pork sausage), the extract obtained from green Robusta coffee showed better results, with the tested concentration of 0.32% being the most promising. Therefore, the Robusta green coffee extract was a suitable alternative for sodium erythorbate replacement in fresh pork sausage. Moreover, Robusta green extract—a natural antioxidant—applied to fresh pork sausage ensured stability against lipid oxidation and generated sausages that were sensorially accepted by consumers.

## Figures and Tables

**Table 1:** Bioactive compounds and antioxidant activity in Arabica coffee extracts obtained by ultrasound-assisted hydroalcoholic extraction using a central composite rotational design 2^3^ with triplicate at the central point.

Experiment No.	Extraction Conditions Real (Code) Levels ^1^			Bioactive Compounds ^2^			Antioxidant Activity ^2^		
	x_1_	x_2_	x_3_	Y_1_	Y_2_	Y_3_	Y_4_	Y_5_	Y_6_
	(min)	(%)	g/mL	(AU)	(mg/ 100 g)	(mg/ 100 g)	(mg/ 100 g)	(g GAE/ 100 g)	(g TE/ 100 g)
1	5 (−1)	25 (−1)	5/100 (−1)	0.331± 0.004	1401.46 ± 71.44	175.14 ± 13.03	679.24 ± 42.71	5.24 ± 0.09	11.82 ± 0.89
2	15 (1)	25 (−1)	5/100 (−1)	0.352 ± 0.005	1379.94 ± 108.22	163.37 ± 13.55	681.08 ± 22.22	5.02 ± 0.15	11.14 ± 0.74
3	5 (−1)	35 (1)	5/100 (−1)	0.748 ± 0.003	1368.91 ± 35.73	162.86 ± 8.18	607.23 ± 49.75	5.61 ± 0.12	11.06 ± 0.69
4	15 (1)	35 (1)	5/100 (−1)	0.778 ± 0.005	1490.01 ± 157.07	190.16 ± 2.70	971.09 ± 45.58	6.28 ± 0.12	14.56 ± 0.62
5	5 (−1)	25 (−1)	15/100 (1)	0.332 ± 0.004	1302.00 ± 117.03	160.13 ± 5.72	626.33 ± 44.17	4.39 ± 0.06	10.58 ± 0.17
6	15 (1)	25 (−1)	15/100 (1)	0.321 ± 0.004	1281.87 ± 33.94	165.19 ± 7.36	633.38 ± 61.83	4.74 ± 0.05	13.78 ± 0.25
7	5 (−1)	35 (1)	15/100 (1)	0.330 ± 0.004	1267.25 ± 29.55	169.94 ± 8.87	740.82 ± 59.29	4.73 ± 0.09	8.17 ± 0.25
8	15 (1)	35 (1)	15/100 (1)	0.312 ± 0.003	1303.71 ± 19.19	182.53 ± 16.41	628.33 ± 27.80	4.90 ± 0.03	8.26 ± 0.22
9	1.6 (−1.68)	30 (0)	10/100 (0)	0.503 ± 0.004	1298.55 ± 24.55	139.40 ± 1.91	625.51 ± 40.95	4.81 ± 0.05	8.81 ± 0.27
10	18.4 (1.68)	30 (0)	10/100 (0)	0.506 ± 0.004	1442.32 ± 163.96	167.06 ± 2.08	699.77 ± 12.21	5.14 ± 0.07	9.70 ± 0.24
11	10 (0)	21.6 (−1.68)	10/100 (0)	0.236 ± 0.003	1241.14 ± 28.10	137.90 ± 5.43	503.90 ± 33.28	4.14 ± 0.07	6.61 ± 0.39
12	10 (0)	38.4 (1.68)	10/100 (0)	0.531 ± 0.004	1427.44 ± 144.76	165.75 ± 16.10	717.63 ± 75.48	5.39 ± 0.07	9.07 ± 0.27
13	10 (0)	30 (0)	1.6/100 (−1.68)	0.473 ± 0.005	1362.71 ± 24.86	137.15 ± 5.82	688.36 ± 45.36	7.22 ± 0.31	21.37 ± 1.10
14	10 (0)	30 (0)	18.4/100 (1.68)	0.290 ± 0.003	1254.17 ± 47.99	123.14 ± 2.84	647.21 ± 9.60	4.51 ± 0.04	7.96 ± 1.9
15	10 (0)	30 (0)	10/100 (0)	0.408 ± 0.003	1485.43 ± 168.88	263.78 ± 7.89	1309.40 ± 14.45	4.72 ± 0.08	8.58 ± 0.13
16	10 (0)	30 (0)	10/100 (0)	0.408 ± 0.004	1502.14 ± 92.59	269.66 ± 3.78	1254.85 ± 92.98	4.77 ± 0.08	8.16 ± 0.16
17	10 (0)	30 (0)	10/100 (0)	0.406 ± 0.003	1358.34 ± 130.73	266.72 ± 5.84	1282.12 ± 71.60	4.79 ± 0.00	8.45 ± 0.23

^1^ x_1_: ultrasound exposure time; x_2_: ethanol concentration; x_3_: sample—solvent ratio. ^2^ Y_1_: browned compounds; Y_2_: caffeine; Y_3_: 5-caffeoilquinic acid; Y_4_: total chlorogenic acids; Y_5_: Antioxidant activity by Folin–Ciocalteau; Y_6_: ABTS•+ scavenging activity; GAE: gallic acid equivalent; TE: Trolox equivalent. Values are expressed as mean ± standard deviation (*n* = 4 for Y_2_, Y_3_, and Y_4_ and *n* = 9 for Y_1_, Y_5_, and Y_6_, in dry matter).

**Table 2:** Estimated effects for process yield response variables.

Parameter ^1^	Effect	Standard Error	t (7)	*p*-Value	Effect	Standard Error	t (7)	*p*-Value
	Browned compounds				Total chlorogenic acids			
Mean	0.004	0.025	16.210	0.000	1276.202	53.313	23.938	0.000
x_1_	0.075	0.024	0.180	0.862	56.434	50.102	1.126	0.297
x_2_	0.194	0.026	2.894	0.023	−399.337	55.196	−7.235	0.000
x_3_	−0.011	0.024	8.256	0.000	100.625	50.102	2.008	0.085
x_1_^2^	−0.179	0.026	−0.421	0.686	−436.096	55.196	−7.901	0.000
x_2_^2^	−0.012	0.024	−7.606	0.000	−55.538	50.102	−1.108	0.304
x_3_^2^	0.001	0.026	−0.468	0.654	−395.688	55.196	−7.169	0.000
x_1_x_2_	−0.020	0.031	0.020	0.985	60.621	65.433	0.926	0.385
x_1_x_3_	−0.214	0.031	−0.650	0.536	−117.783	65.433	−1.800	0.115
x_2_x_3_	0.004	0.031	−6.943	0.000	−27.139	65.433	−0.415	0.691
	Caffeine				Antioxidant activity by Folin–Ciocalteau			
Mean	1447.391	35.876	40.344	0.000	4.765	0.183	26.078	0.000
x_1_	52.396	33.715	1.554	0.164	0.222	0.172	1.294	0.237
x_2_	−47.090	37.143	−1.268	0.245	0.101	0.189	0.534	0.610
x_3_	55.350	33.715	1.642	0.145	0.620	0.172	3.610	0.009
x_1_^2^	−72.704	37.143	−1.957	0.091	−0.048	0.189	−0.255	0.806
x_2_^2^	−97.890	33.715	−2.903	0.023	−1.163	0.172	−6.773	0.000
x_3_^2^	−91.023	37.143	−2.451	0.044	0.734	0.189	3.879	0.006
x_1_x_2_	49.801	44.031	1.131	0.295	0.182	0.224	0.811	0.444
x_1_x_3_	−20.811	44.031	−0.473	0.651	0.017	0.224	0.078	0.940
x_2_x_3_	−22.605	44.031	−0.513	0.623	−0.284	0.224	−1.265	0.246
	5-caffeoilquinic acid				ABTS•+ scavenging activity			
Mean	264.471	13.546	19.525	0.000	8.367	1.456	5.747	0.001
x_1_	11.674	12.730	0.917	0.390	1.118	1.368	0.817	0.441
x_2_	−65.357	14.024	−4.660	0.002	0.808	1.507	0.536	0.609
x_3_	12.965	12.730	1.018	0.342	−0.165	1.368	−0.121	0.907
x_1_^2^	−66.357	14.024	−4.732	0.002	−0.196	1.507	−0.130	0.900
x_2_^2^	−5.464	12.730	−0.429	0.681	−4.442	1.368	−3.247	0.014
x_3_^2^	−81.717	14.024	−5.827	0.001	4.641	1.507	3.079	0.018
x_1_x_2_	11.645	16.625	0.700	0.506	0.268	1.787	0.150	0.885
x_1_x_3_	0.529	16.625	0.032	0.976	0.118	1.787	0.066	0.949
x_2_x_3_	3.162	16.625	0.190	0.855	−2.648	1.787	−1.482	0.182

^1^ x_1_: ultrasound exposure time; x_2_: ethanol concentration; x_3_: sample—solvent ratio.

**Table 3:** ANOVA of models.

Parameter	Factor Variable	Sum of Squares	Degree of Freedom	Mean Square	F	F_critical_	*p*-Value
Browned compounds	Regression	0.351	5	0.070	52.746	3.204	0.000
	Residue	0.015	11	0.001			
	Total	0.366	16	0.023			
Caffeine	Regression	87,551.36	7	12,507.34	3.877	3.293	0.031
	Residue	29,030.81	9	3225.65			
	Total	116,582	16	7286.39			
Acid 5-caffeoylquinic	Regression	28,944.07	7	4134.87	9.567	3.293	0.002
	Residue	3889.89	9	432.21			
	Total	32,833.96	16	2052.12			
Total chlorogenic acids	Regression	981,445.44	7	140,206.49	18.351	3.293	0.000
	Residue	68,763.91	9	7640.32			
	Total	1,050,208.35	16	65,638.02			
Antioxidant activity (Folin–Ciocalteau)	Regression	7.94516	5	1.589	21.357	3.204	0.000
	Residue	0.818425	11	0.074			
	Total	8.763587	16	0.548			
ABTS•+ scavenging activity	Regression	151.45410	5	30.291	7.047	3.204	0.004
	Residue	47.2831	11	4.298			
	Total	198.7372	16	12.421			

**Table 4:** Characterization of coffee bioactive compounds and antioxidant activity extracted under optimized conditions and on the central point.

Sample ^1^	Browned Compounds (UA) ^2^	Caffeine (mg/100 g) ^2^	5-Caffeoylquinic (mg/100 g) ^2^	Folin–Ciocalteau (g EAG/100 g) ^2^	ABTS+• (g Eq Trolox/100 g) ^2^
ORR	0.364 ± 0.013 ^b^	1881.8 ± 18.8 ^c^	482.9 ± 21.6 ^c^	8.66 ± 0.14 ^b^	16.28 ± 1.00 ^b^
OAR	0.505 ± 0.035 ^a^	1652.6 ± 47.4 ^d^	703.8 ± 38.3 ^c^	4.29 ± 0.19 ^g^	12.09 ± 0.38 ^d^
CRR	0.303 ± 0.016 ^c^	1764.1 ± 34,8 ^d^	427.3 ± 8.1 ^c^	7.36 ± 0.32 ^d^	15.28 ± 1.47 ^bc^
CAR	0.379 ± 0.018 ^b^	1244.9 ± 52.0 ^e^	464.5 ± 35.7 ^c^	3.41 ± 0.14 ^h^	11.61 ± 1.22 ^d^
ORG	-	3148.1 ± 13.5 ^a^	6706.4 ± 23.5 ^a^	9.54 ± 0.40 ^a^	14.52 ± 1.16 ^c^
OAG	-	2145.0 ± 106.9 ^b^	5439.6 ± 393.1 ^b^	6.35 ± 0.20 ^e^	20.54 ± 1.04 ^a^
CRG	-	3114.8 ± 50.0 ^a^	6417.1 ± 22.0 ^a^	8.04 ± 0.14 ^c^	12.93 ± 0.78 ^d^
CAG	-	2210.8 ± 43.9 ^b^	5286.8 ± 259.5 ^b^	5.72 ± 0.17 ^f^	15.31 ± 0.34 ^bc^
Antioxidant					
DORR	-	-	-	27.25 ± 0.16	41.35 ± 1.12
DORG	-	-	-	24.79 ± 0.84	42.93 ± 1.57
SE	-	-	-	80.01 ± 0.46	124.70 ± 0.92

^1^ ORR: medium roast Robusta coffee with extraction at the optimum point; OAR: medium roast Arabica coffee with extraction at the optimum point; ORG: green Robusta with extraction at the optimum point; OAG: medium roast Arabica with extraction at the optimum point; CRR: medium roast Robusta with extraction at the central point; CAR: medium roast Arabica with extraction at the central point; CRG: green Robusta with extraction at the central point; CAG: medium roast Arabica with extraction at the central point; DORR: dried medium roast Robusta with extraction at the optimum point; DORG: dried green Robusta with extraction at the optimum point; SE: sodium erythorbate. ^2^ Values are expressed as mean ± standard deviation (*n* = 9) in dry matter. Mean values followed by different letters indicate a significant difference in the same column (Tukey test, *p* ≤ 0.05).

**Table 5:** Physicochemical and sensory acceptance parameters of fresh pork sausage added with Robusta coffee, green and medium roasted, dry extract, and control added with sodium erythorbate.

Sample ^1^	TBARs (mg of Malonaldehyde/kg of Sample) (*n* = 3)			Sensory Acceptance ^2^ (*n* = 62)						
	0 Day	10 Day	20 Day	Smell ^3^	Color ^3^	Flavor ^3^	Texture ^3^	Global Acceptance ^3^	Purchase Intention ^4^	
GDE1%	0.681 ± 0.056 ^Aa^	0.696 ± 0.059 ^Aa^	0.677 ± 0.068 ^Aa^	6.76 ± 1.50 ^c^	7.19 ± 1.39 ^b^	7.16 ± 1.43 ^bc^	7.32 ± 1.56 ^a^	7.34 ± 1.09 ^bc^	3.85 ± 0.91 ^bc^	
GDE032%	0.612 ± 0.056 ^Aa^	0.595 ± 0.071 ^Aa^	0.577 ± 0.079 ^Aa^	7.43 ± 1.16 ^ab^	7.71 ± 1.05 ^ab^	7.63 ± 1.37 ^ab^	7.68 ± 1.19 ^a^	7.77 ± 1.07 ^ab^	4.21 ± 0.86 ^ab^	
C	0.662 ± 0.038 ^Aa^	0.697 ± 0.065 ^Aa^	0.628 ± 0.048 ^Aa^	7.81 ± 1.24 ^a^	8.00 ± 0.88 ^a^	8.19 ± 1.01 ^a^	7.92 ± 1.37 ^a^	8.19 ± 0.91 ^a^	4.53 ± 0.98 ^a^	
RDE032%	0.671 ± 0.056 ^Aa^	0.671 ± 0.049 ^Aa^	0.687 ± 0.067 ^Aa^	6.84 ± 1.73 ^bc^	6.15 ± 1.94 ^c^	6.92 ± 1.85 ^c^	7.31 ± 1.56 ^a^	7.03 ± 1.48 ^c^	3.66 ± 1.07 ^c^	
RDE1%	0.655 ± 0.054 ^Ab^	0.687 ± 0.045 ^Ab^	0.644 ± 0.071 ^Ab^	6.84 ± 1.73 ^bc^	6.84 ± 1.73 ^bc^	6.84 ± 1.73 ^bc^	6.84 ± 1.73 ^bc^	6.84 ± 1.73 ^bc^	6.84 ± 1.73 ^bc^	
	**Texture parameters (*n* = 3) ^2^**				**Color parameters of raw sausages (*n* = 3) ^2^**			**Color parameters of baked** **sausages (*n* = 3) ^2^**		
	**Hardness** **(N)**	**Elasticity** **(mm)**	**Chewiness** **(N/mm)**	**Cohesiveness**	L*	a*	b*	L*	a*	b*
GDE1%	44.55 ± 3.37 ^a^	0.843 ± 0.008 ^a^	23.79 ± 3.06 ^a^	0.551 ± 0.048 ^c^	55.07 ± 2.92 ^ab^	14.85 ± 0.90 ^a^	14.77 ± 0.07 ^b^	60.58 ± 0.90 ^a^	9.91 ± 0.96 ^b^	14.07 ± 0.70 ^b^
GDE032%	47.79 ± 4.23 ^a^	0.854 ± 0.020 ^a^	25.23 ± 0.56 ^a^	0.505 ± 0.005 ^c^	59.54 ± 0.87 ^a^	8.59 ± 1.64 ^c^	11.66 ± 0.30 ^c^	60.63 ± 1.23 ^a^	13.04 ± 0.27 ^a^	11.19 ± 0.71 ^c^
C	42.38 ± 0.87 ^a^	0.892 ± 0.003 ^a^	22.35 ± 1.02 ^a^	0.581 ± 0.007 ^bc^	58.06 ± 1.83 ^a^	14.49 ± 0.90 ^a^	10.87 ± 0.69 ^c^	60.99 ± 1.33 ^a^	14.87 ± 0.41 ^a^	10.64 ± 0.30 ^c^
RDE032%	47.37 ± 1.24 ^a^	0.826 ± 0.037 ^a^	25.58 ± 1.04 ^a^	0.663 ± 0.039 ^ab^	51.91 ± 1.02 ^b^	12.58 ± 0.46 ^ab^	17.96 ± 0.62 ^a^	49.31 ± 2.60 ^b^	9.97 ± 0.75 ^b^	17.67 ± 0.20 ^a^
RDE1%	46.63 ± 1.28 ^a^	0.843 ± 0.027 ^a^	23.74 ± 0.30 ^a^	0.694 ± 0.036 ^a^	40.58 ± 1.37 ^c^	11.67 ± 0.50 ^b^	16.74 ± 1.22 ^ab^	40.31 ± 0.68 ^c^	9.33 ± 0.33 ^b^	14.3 ± 0.30 ^b^

^1^ Samples: GDE1%: fresh pork sausage with 1% green RC dry extract; GDE032%: 0.32% green RC dry extract; C: Control (with sodium erythorbate); RDE1%: 1% medium roast dry extract; RDE032%: 0.32% medium roast RC dry extract. ^2^ Results expressed as mean ± standard deviation; means with different superscript lowercase letters between the lines and uppercase letters between the columns indicate a significant difference using the Tukey test (*p* ≤ 0.05). ^3^ 9-point hedonic scale (1 = extremely disliked and 9 = extremely liked. ^4^ 5-point structured scale (1 = would certainly not buy and 5 = would certainly buy).

## Data Availability

The original contributions presented in the study are included in the article. Further inquiries can be directed to the corresponding author.
